# CYP2S1 rs338599 polymorphism confers reduced risk to anti‐tuberculosis drug‐induced liver injury and may be a novel marker for its risk prediction

**DOI:** 10.1002/jcla.24478

**Published:** 2022-05-09

**Authors:** Liyuan Wang, Hui Wang, Ying Qi

**Affiliations:** ^1^ 612973 Department of Tuberculosis Affiliated Hospital of Hebei University Baoding China

**Keywords:** anti‐tuberculosis drug‐induced liver injury, CYP2S1, gene polymorphism, novel marker, risk prediction

## Abstract

**Purpose:**

In the present study, we would like to explore whether Cytochrome P450 2S1 (CYP2S1) rs338599 polymorphism confers risk to anti‐tuberculosis drug‐induced liver injury (ADLI) and provide evidence of being used as novel marker for ADLI risk prediction.

**Patients and methods:**

A total of 162 pulmonary tuberculosis patients admitted to Affiliated Hospital of Hebei University from August 2018 to March 2021 were selected. Patients who developed into ADLI were assigned as ADLI group (*n* = 50), and those who did not developed into ADLI were assigned as non‐ADLI group (*n* = 112). The CYP2S1 rs338599 polymorphism was detected by polymerase chain reaction‐restriction fragment length polymorphism (PCR‐RFLP) method using binary logistic regression analyses through adjusting for age and sex.

**Results:**

No difference was detected in age, sex, smoking status, profession, education level, marital status, alcohol consumption, or using liver‐protecting drugs (*p* > 0.05). Compared with non‐ADLI group, GG genotype and G allele were significantly higher in ADLI group (*p* < 0.05).

**Conclusion:**

Our results indicated that CYP2S1 rs338599 polymorphism conferred reduced risk to ADLI. The tuberculosis patients who had GG genotype or G allele were not susceptible to ADLI. CYP2S1 rs338599 polymorphism may be a novel marker for ADLI risk prediction.

## Graphical abstract

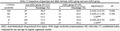

## 1 INTRODUCTION

Since the 21st century, the incidence of tuberculosis in China has been reduced to 50% at the beginning of this century, but there are still 1 million new tuberculosis patients every year.^1^, ^2^, ^3^ At present, the standard drugs for anti‐tuberculosis treatment are isoniazid, rifampicin, pyrazinamide combined with ethambutol, or streptomycin. Among them, isoniazid, rifampicin, and pyrazinamide have potential hepatotoxicity, and the incidence of adverse liver reactions is about 2.55%–11.9%,^4^ ^,^ ^5^ which may lead to liver failure and death in severe cases. Anti‐tuberculosis drug‐induced liver injury (ADLI) is one of the important reasons for drug failure and subsequent drug resistance of tuberculosis. However, ADLI has the characteristics of obvious individual differences and unpredictability, which brings great health hazards and economic losses to patients and increases the medical burden.

Non‐genetic factors such as gender, age, alcohol consumption, history of liver disease, concomitant infection, and nutritional status are risk factors for ADLI, but their clinical prediction is limited.^6^ Existing studies have found that genetic factors are one of the key factors for individual differences in ADLI.^6^ There are two possible mechanisms of ADLI. First, abnormal metabolism of drugs leads to accumulation of toxic products, resulting in intracellular oxidative stress response and changes in mitochondrial permeability, and then leading to apoptosis or necrosis of liver cells.^7^ Second, drugs cause immune regulation and inflammatory responses in the liver.^8^

Drug metabolism enzymes (DME) play a significant role in drug detoxification and activation, which exert important effect on drug efficacy and sensitivity to toxicity. Anti‐tuberculosis drugs are mainly metabolized by two kinds of DMEs including phase I enzymes and phase II enzymes. Cytochrome P450 2S1 (CYP2S1) is a family monooxygenase, which plays an important role in the metabolism of various substances including Anti‐tuberculosis drugs. The rs338599 loci have been most important and most widely studied of CYP2S1 gene polymorphism. In the present study, we would like to explore whether CYP2S1 rs338599 polymorphism at the respective gene loci confers risk to ADLI and provide evidence of being used as novel marker for ADLI risk prediction.

## 2 MATERIALS AND METHODS

### 2.1 Patients and controls

One hundred and sixty‐two tuberculosis patients who were treated at the outpatient department or inpatient department of Affiliated Hospital of Hebei University were prospectively enrolled. During the study period, anti‐tuberculosis drugs were taken. Based on whether it develops into liver injury, tuberculosis patients were divided into ADLI group (50 patients) and non‐ADLI group (112 patients). Inclusion criteria were as follows: all patients who were treated with first‐line anti‐tuberculosis drugs for 6 months; (2) all patients were newly diagnosed tuberculosis patients. Exclusion criteria were as follows: patients who were suffering from viral hepatitis, alcoholic liver disease or autoimmune hepatitis; patients who were suffering from abnormal liver function before anti‐tuberculosis treatment. This study was approved by the Medical Ethics Committee of Affiliated Hospital of Hebei University. All the tuberculosis patients provided informed consent. This investigation was conducted based on the principles of the declaration of Helsinki.

### 2.2 Epidemiological investigation

During the study period, all tuberculosis patients’ general condition and basic information should be recorded in detail through an epidemiological investigation. The epidemiological survey consisted of age, sex, body mass index (BMI), educational qualifications, marital status, profession, smoking status, alcohol consumption, medical history, and liver function. The survey method standard for smoking and drinking was recommended by World Health Organization (1984). Objective indicators should be used as far as possible, and two pre‐surveys should be conducted before the formal survey performed. During the study period, patients in the non‐ADLI group were followed up for recent regular liver function tests.

### 2.3 CYP2S1 gene polymorphism

Three militer of fasting venous blood was collected from all tuberculosis patients, which was immediately centrifuged and stored at −80°C for detection. DNA extraction was performed using human peripheral blood genomic DNA Extraction and Purification Kit (Zhongke Leiming Biotechnology Co., LTD). The selection SNP loci were based on searching bioinformatics databases and previous literatures. The genotypes of CYP2S1 gene polymorphism were performed using the polymerase chain reaction–restriction fragment length polymorphism (PCR‐RFLP) method. The representative PCR‐amplified DNA samples were examined by DNA sequencing in order to confirm the genotyping results. The detailed primer sequence for the present polymorphism was as follows: Forward: 5′‐CTCCTGATCTCAGGTTCTGAAGG‐3′, Reverse: 5′‐CAGGGGTAGTCCTGGGTGTA‐3′.

### 2.4 Statistical analysis

The SPSS 19.0 was applied for whole analysis, the measurement data were represented by (X ± S), and the comparison of the means of two independent samples was performed by t test. The counting data were expressed as percentage (%) and was performed by chi‐square test. Hardy–Weinberg equilibrium (HWE) among case group and control group were checked with the goodness‐of‐fit chi‐square test. Odds ratios (OR) and 95% confidence intervals (CI) were counted to evaluate the relationship between risk factors and ADLI by performing binary logistic regression. Both age and sex were adjusted to assess the relative risk. *p* < 0.05 indicated the difference was statistically significant.

## 3 RESULTS

### 3.1 General information of tuberculosis patients

No difference was detected in age, sex, smoking status, profession, education level, marital status, alcohol consumption, or using liver‐protecting drugs (*p* > 0.05). The detailed information is shown in Table 1. Furthermore, no difference was detected in five anti‐tuberculosis drugs. The detailed information is shown in Table 2.

**TABLE 1:** Basic information of both ADLI group and non‐ADLI group

Basic information		Non‐ADLI (*N =* 112)	ADLI (*N* = 50)	*p*
Age		58.60 ± 10.00	57.40 ± 9.41	0.461
Sex	Male	77	36	0.715
	Female	35	14	
Smoking status	Yes	60	29	0.613
	No	52	21	
Alcohol Consumption	Yes	42	18	0.862
	No	80	32	
BMI	BMI < 18.5 kg//m^2^	23	10	0.664
	BMI≥18.5 kg//m^2^	89	40	
Education	Senior	34	15	0.945
	Junior	52	20	
	Primary	36	16	
Profession	Worker	36	17	0.896
	Farmer	50	16	
	Others	36	17	
Marital status	Married	92	47	0.052
	Unmarried	20	3	
Liver‐protecting drugs	Use	89	42	0.666
	Unused	23	8	

Abbreviations: ADLI, anti‐tuberculosis drug‐induced liver injury; BMI, body mass index.

**TABLE 2:** Comparison of drugs information of both ADLI group and non‐ADLI group

Drugs information		Non‐ADLI (*N* = 112)	ADLI (*N* = 50)	*p*
Isoniazid	Yes	23	10	0.664
	No	89	40	
Rifampicin	Yes	85	39	0.843
	No	27	11	
Pyrazinamide	Yes	42	18	0.862
	No	80	32	
Ethambutol	Yes	23	10	0.664
	No	89	40	
Streptomycin	Yes	20	9	1.000
	No	92	41	

Abbreviations: ADLI, Anti‐tuberculosis drug‐induced liver injury; BMI, body mass index.

### 3.2 CYP2S1 gene polymorphism

Polymerase chain reaction‐restriction fragment length polymorphism method showed that compared with non‐ADLI group, GG genotype and G allele were significantly higher in ADLI group (*p* < 0.05). The detailed information is shown in Table 3.

**TABLE 3:** Comparison of genotype and allele between ADLI group and non‐ADLI group

CYP2S1 rs338599	Non‐ADLI group (*N* = 112)		ADLI group (*N* = 50)		OR (95% CI)s^a^	*p* ^a^
	*n*	Percentage (%)	*n*	Percentage (%)		
GG	65	58.0	19	38.0	0.31(0.13–0.73)	0.006
GC	30	26.8	15	30.0	0.53(0.21–1.34)	0.180
CC	17	15.2	16	32.0	1.00^REF^	
G	160	71.4	53	54.8	0.45(0.28–0.73)	0.001
C	64	28.6	47	45.2	1.00^REF^	

Abbreviations: ADLI, anti‐tuberculosis drug‐induced liver injury; CI, confidential index; OR, odds ratio; SNP, single‐nucleotide polymorphism.

^a^ Adjusted for sex and age by logistic regression model.

## 4 DISCUSSION

Drug‐induced liver injury is one of the most common and serious adverse drug reactions, which is caused by drug hypersensitivity or their metabolites during drug‐use process. Drug‐induced liver injury leads to 3%–5% jaundice, and it is also the main cause of acute liver failure, which can lead to death in severe cases.^9^ Currently, more than 1,100 drugs are known to have potential hepatotoxicity, including Chinese herbal medicines, anti‐tuberculosis drugs, anti‐infective drugs, antipyretic analgesics, and anti‐tumor drugs.^4^ In Chinese population, liver injuries caused by anti‐tuberculosis drugs are mainly isoniazid, rifampicin, and pyrazinamide, which accounts for 21.56% of all drug‐induced liver injuries.^5^

Human CYP2S1 gene is 13 kb in length, located on chromosome 19q. 13.2, including 9 exons, encoding 504 amino acid proteins with molecular weight of about 55.8 kDa, with 8 single‐nucleotide polymorphisms (SNPs) loci. CYP2S1 mRNA expression level is low in liver, but high in extrahepatic tissues such as respiratory and digestive systems.^10^ In addition, CYP2S1 mRNA and protein are also expressed in human skin. CYP2S1 plays an important physiological role in the metabolism of dodecanoids, and may play a role in inflammatory response, tumor, and other pathological processes.^11^

Domestic and foreign studies have found that one‐phase DME CYP2E1,^12^ two‐phase DME NAT2,^13^ GSTM1,^14^ three‐phase drug transporter ABCB1,^15^ immune regulatory genes HLA‐DQA1, and HLA‐DQB1^16^ were significantly correlated with liver injury induced by anti‐tuberculosis drugs. These studies examined the contribution of genetic factors to liver injury induced by anti‐tuberculosis drugs. As far as we know, this is the first study, which investigates the CYP2S1 rs338599 polymorphism in ADLI risk. Our results indicated that CYP2S1 rs338599 polymorphism conferred decreases risk to ADLI. The tuberculosis patients who had GG genotype or G allele were not susceptible to ADLI. In other words, CYP2S1 rs338599 polymorphism had protective effect on ADLI.

In the present study, there were two potential limitations must be acknowledged. Firstly, all subjects were enrolled from only one hospital and all patients came from Hebei population. China is a country with vast territory and 56 ethnic groups. In addition, race, ethnicity, and region have important and profound influence on gene polymorphism. The current results do not necessarily apply elsewhere. Secondly, current study only investigated only one SNP. It would be interesting and meaningful to research more SNP loci of CYP2S1 gene to learn about their associations with ADLI risk. Therefore, current results must be treated and interpreted with caution due to objectives and limitations.

## 5 CONCLUSION

Our results indicated that CYP2S1 rs338599 polymorphism conferred reduced risk to ADLI. The tuberculosis patients who had GG genotype or G allele were not susceptible to ADLI. CYP2S1 rs338599 polymorphism may be a novel marker for ADLI risk prediction.

## AUTHOR CONTRIBUTIONS

Ying Qi conceived study design. Liyuan Wang conceived the content concept. Liyuan Wang and Hui Wang performed the data collection, extraction, analyzed the data, interpreted, and reviewed the data and drafts. Ying Qi reviewed the final draft. All authors were involved in literature search, writing the article, and had final approval of the submitted and published versions; contributed to data analysis, drafting, or revising the article; have agreed on the journal to which the article will be submitted; gave final approval of the version to be published; and agree to be accountable for all aspects of the work.

## CONFLICT OF INTEREST

The authors declare that they have no competing interests.

## Data Availability

All data generated or analyzed during this study are included in this published article.
